# Young users of social media: an analysis from a gender perspective

**DOI:** 10.3389/fpsyg.2024.1375983

**Published:** 2024-05-30

**Authors:** Sue Aran-Ramspott, Oihane Korres-Alonso, Iciar Elexpuru-Albizuri, Álvaro Moro-Inchaurtieta, Ignacio Bergillos-García

**Affiliations:** ^1^Blanquerna School of Communication and International Relations, Ramon Llull University, Barcelona, Spain; ^2^Faculty of Education and Sport, University of Deusto, Donostia, Spain; ^3^Faculty of Education and Sport, University of Deusto, Bilbao, Spain; ^4^CESAG, Comillas Pontifical University, Mallorca, Spain

**Keywords:** gender, social media, adolescence, youth, edu-communication, empowerment, perception, media literacy

## Abstract

One of the major challenges for edu-communication research is to analyze the influence of social media on young and adolescent users. This article examines the evaluation of gender inequalities – real and symbolic – in the consumption of social networks such as YouTube and Instagram among young people. Within the framework of a Research & Development & innovation (R&D + *i*) project, it presents a discursive-theoretical analysis of how young users of social media perceive the presence and representation of gender on social media and whether such digital representations can be associated with an empowering gender perspective. This study presents results from 14 focus groups (*N* = 83), composed of students aged 12 to 18, drawn from three Spanish Autonomous Communities (Catalonia, the Balearic Islands and the Basque Country). The results show that gender issues arise in participants’ conversations, especially among female participants, who perceive the importance of physical appearance on platforms such as Instagram and TikTok. Female participants feel more pressure in terms of appearance and dress compared to male participants. Among male participants there are more expressions of self-affirmation and more mentions related to fun and social prestige. Both male and female participants express concern about the impact of that pressure on younger girls. The influence of social media on self-image is more evident among female participants, who make frequent mention of the importance of self-esteem in relation to beauty standards and exposure to idealized body images. Notably, there were no comments by male participants that acknowledge any influence of social media on their self-image. The findings are in line with existing research and taken as a whole gives rise to concern as to the gender disparities observed in the use of social media, which do not constitute a picture of female empowerment. This research underlines the importance of promoting a respectful and equitable environment in relation to gender equality within digital spaces. Thus, this study provides support for the need to develop and implement edu-communicative initiatives to foster critical thinking around the influence of social media in this context and the evaluation of the impact of such initiatives in future research.

## 1 Introduction

One of the major challenges for research in edu-communication – a term used in the field of Communication (Orozco, 1997, 2010; Kaplún, 2013) and which UNESCO defined in 2002 as teaching and critical learning about the media – and its dissemination to the wider community is to analyze the influence of social media on young users. Prior research has indicated the significant impact of social media on identity development (Ahn, 2011; Valkenburg and Peter, 2011; boyd, 2014; Baym, 2018; van Eldik et al., 2019). Social media such as YouTube and Instagram are considered privileged spaces for the construction and (self-)representation of youth identity (Cover, 2012; Thumim and Enli, 2012; boyd, 2014), and allow for parasocial relationships with influencers (Rihl and Wegenerpp, 2017; Ferchaud et al., 2018; Rasmussen, 2018), who may act as role models (Westenberg, 2016). As studies in Spain and internationally show, adolescents are now heavy users of social media use, especially YouTube and Instagram (Ríos Hernández et al., 2022; IAB, 2023). Here, however, we are concerned with the extent to which this scenario of hyperconnectivity favors or impedes the (co-)responsibility of the new generations for gender equality in the digital media space, as new moral subjects – active audiences (Ruiz, 2015). That responsibility is linked to the concept of participation that we take up here from the perspective of the complexity and ambivalences that its practice requires (Jenkins and Carpentier, 2013), so as to overcome the magical or cosmetic roles often assigned to that have often been attributed to the participatory activity of audiences (Bergillos, 2019).

As is well known, digital platforms are part of the societal and media discourse that constitutes and shapes collective imaginaries, including representations of gender. Since the 1970s, the incorporation of gender into research approaches has highlighted stereotypical representations of men and women (Bernárdez, 2015) that lead people to internalize inequality in ways that even today continue to hinder the development of fairer societies.

Progress toward equality and women’s empowerment has been marked by a number of milestones on a global scale, such as the Fourth World Conference on Women in Beijing in 1995. Within the European Union there has been progress at a political level such as the Charter of Fundamental Rights, the creation of the European Institute for Gender Equality and the issue of the Gender Equality Strategy by the European Commission (García-Ruiz et al., 2014). In fact, gender equality and empowering all women and girls is one of the UN’s 17 Sustainable Development Goals and is integral to every facet of inclusive, sustainable development. As stated by UN Women (2023), there is an urgent need to identify and eliminate all forms of discrimination against women and girls, in both the public and the private sphere. In Spain, it is a cause for concern that the progress made in bridging the gender gap since 2006 (IME, 2023) will come under threat between 2021 and 2023 (Statista, 2023), at the same time as the country is seeing growing anti-feminism and denial of gender violence among adolescent males (Boneta-Sádaba et al., 2023).

In the digital space, the rise in hate speech aimed at feminist principles or directly against women, has been noted, as shown by different assessments of the internet and social media (EIU, 2021; Tortajada and Vera, 2021). Several studies show how users of social media tend to coalesce around highly polarized positions driven by partisan differences in the framing of discourse (Demszky et al., 2019). As Diepeveen (2024, p. 5) points out, “over the past ten years, online spaces with content that rejects feminism and gender equality and promotes male supremacy – sometimes termed the “manosphere” (Marwick and Caplan, 2018; Kimeu, 2023) have become increasingly prevalent.” Some authors (Medina and Talarn, 2020, pp. 494–495) observe the spread of a neo-liberal feminism among young men and women, “the belief that the acceptance of rigid patterns of an idealized femininity is, in fact, an exercise of free and determined will.” Among the risks of social media consumption among young people, the impact of esthetic standards from the world of fashion and the persistence of stereotyped roles are highlighted (Fernández-de-Arroyabe-Olaortua et al., 2018). Nevertheless, there are other more encouraging developments. The notoriety of popular feminism (Banet-Weiser, 2018) seems to have influenced the digital imaginary around the construction of gender, giving rise to more diverse and inclusive representations that partially invalidate postfeminist theses (Caballero-Gálvez et al., 2017; Keller and Ryan, 2018). The opportunities that the consumption of digital sources can offer compared to the use of other media are said to include less divergence in the roles assigned to males and females (Feijoo and García-González, 2017). In Spain, according to the Youth Report (Injuve, 2021), young people have an interest in gender inequality, very possibly in consequence of having been socialized in an environment in which the most actively advocated social issues were associated with feminism (Peña-Fernández et al., 2023).

In order to understand the evidence on whether, how and to what extent social media affect gender norms among adolescents, in 2023 the ODI (Diepeveen, 2024) conducted a targeted review of empirical studies published since 2015, focusing on publications in English, Spanish, French and German on adolescent boys. Included in the review were 51 studies on social media platforms with public-facing content, such as Instagram and YouTube. The evidence was diverse. As Diepeveen (2024) summarizes, many of the quantitative studies explored correlations and tended to assume that the direction of influence went from social media platforms to gender norms and attitudes, rather than vice versa or in both directions. Qualitative studies provided a useful corrective, revealing the many ways in which adolescents use social media and select and produce online content in function of their pre-existing interests and attitudes.

Our article provides qualitative research that captures the subtle and sometimes contradictory comments of adolescents themselves about what they perceive social media to be and, in turn, what it is that social media entrench or undermine.

For all these reasons, it appears to us appropriate to address the discourse of adolescents from a gender perspective, in order to determine whether they take gender differences into account in their assessments of social media such as YouTube and Instagram and whether they make any link between those differences and forms of power and discrimination (EIGE, 2023). The gender perspective is (or should be) understood, consequently, as a consubstantial element of the (trans)media education or literacy of digital generations, in line with the understanding of those terms and the importance attributed to them by various authors (García-Ruiz and Pérez-Escoda, 2019; Balladares-Burgos and Jaramillo-Baquerizo, 2022) and international organizations and initiatives such as Unesco, the Agenda 2030 action plan and Unicef. Spaces for reflection, debate and content creation from a feminist perspective can be created for young girls and adolescent women and their male peers through such media empowerment (Tornay-Marquez, 2019).

## 2 Gender perspective and transmedia education

Despite the theoretical nuances between their conceptual frameworks, edu-communication and transmedia education or transmedia literacy can be understood as convergent approaches from the perspectives of education and communication, respectively, to the growing interrelation between media, communication and education in different stages of the lives of children, adolescents and young adults. Both theoretical frameworks aim to encourage and facilitate critical reflection and empowerment of users of media, information, and communication technology.

The concept of media literacy has been approached from different perspectives and has evolved as digital and virtual technology has developed. As Ríos Hernández et al. (2022) point out, in today’s digital ecosystem audiences have a more active role, generating a relationship of dialog between different media and their users. Thus, Scolari (2018) posits the term transmedia literacy to reflect contemporary reality: such literacy consists not only in critical analysis of content, but also in treating consumers of media as active subjects in the digital world, with increasingly sophisticated interpretative and creative skills.

In relation to the media literacy of young people, we are in agreement with theoretical stances that reject simplistic solutions or hypodermic’ processes (Bragg et al., 2011). Zimmerman (2000) expands this holistic approach by emphasizing the positive aspects of human behavior (including identification and capacity building) that accompany “the analysis of the influence of the environment rather than blaming the victims” (Silva and Martínez, 2004, p. 2).

Several formulations have been proposed of the competencies and indicators that make up (trans)media literacy. For example, the dimensions of media competence put forward by Ferrés and Piscitelli (2012): language, technology, processes of interaction, processes of production and dissemination, ideology, and values and esthetics. Similarly, Scolari (2018) posits the following transmedia competencies: production, management, performative, media and technology, narrative and esthetics, risk prevention, and ideologies and ethics.

Our principal interest resides in the competencies that can be seen as making up a journey, since such a journey can bring together the notion of transmedia education – alert to new technological and social realities – with empowerment as a process (Montero, 2003). That is so not only because it is a non-linear model of change (Kabeer, 1999), but also because it is in itself a double process: individual, as the acquisition of greater autonomy, and collective, “with the aim of achieving a fair and egalitarian society, especially in terms of relations between men and women” (Charlier and Caubergs, 2007, p. 6).

Making a link between media literacy and the concept of empowerment raises the issue of the role of men’s and women’s involvement and participation in the digital environment. In academic works, different studies (Tufecki, 2017; Dussel et al., 2021) discuss the contradictions of the new conditions of popular participation in the digital world. While some authors observe that more gender differences have been found in the offline world, in the “real” life of young people (Renau et al., 2012), others continue to focus on the risks presented by beauty standards and the persistence of stereotypical female roles (Fernández-de-Arroyabe-Olaortua et al., 2018; Santos et al., 2022) and stereotypical gender roles in general (Ringrose et al., 2013; Van Oosten et al., 2017). In other words, there may be inequalities in media access and representation that reflect differences in digital participation. We want to determine whether any such inequalities are found among adolescents and young adults: a particularly important stage in the journey of edu-communication and transmedia literacy development.

Previous studies on social media and young people point out that, beyond the interests of each individual, the self-perception of the skills and competencies needed to manage cyberspace is different between males and females, with males more positive (Siddiq and Scherer, 2019; Estanyol et al., 2023). However, there are also studies that point to the influence of sexist stereotypes that can sentence one of the genders to a position of inequality in the “onlife reality”, a term used by Floridi (2015, p. 1) to refer to a hyperconnected reality in which online and offline realities are in practice inseparable [cited by Serrate-González et al. (2023)].

Specifically, we are interested in the perceptions conveyed by male and female participants of their experience on YouTube and Instagram around gender identity in the representations and discourses in these media. To that end, our research questions can be summarized as two questions:

*RQ*1. How do young users of social media such as YouTube and Instagram of either sex see the presence and representation of gender on social media?

*RQ*2. Is it possible to say that the way they see gender on social media is empowering for them?

## 3 Materials and methods

### 3.1 Participants

An exploratory qualitative study was carried out using focus groups (FG) with adolescents aged 12 to 18 in the Autonomous Communities of Catalonia, the Basque Country and the Balearic Islands in Spain according to a criterion of convenience, in light of the location of the three universities participating in the project. The selection of the sample responds to age criteria, to cover the three stages of adolescence (UNICEF, 2024): early or initial adolescence (10–13 years), middle adolescence (14–16) and late or post-adolescence (17 up to as late as 21 years of age), in three geographic areas, and to a criterion of convenience in the selection of the three Autonomous Communities, corresponding in which the three participating universities are located (anonymized). The sample was selected with the help of the educational settings that had participated in a previous questionnaire (Aran-Ramspott et al., 2022) using two filter criteria:

- age of the participants: three categories according to stage of education, first year of Compulsory Secondary Education (approximately 12 years of age); fourth year of Compulsory Secondary Education (15–16 years old) and first year university students (18–20 years old) following Communication and Education courses most closely related to Media literacy at each of the three participating universities.
- gender balance.

Finally, in late 2021 and early 2022, 14 FGs were held, involving a total of to 76 students (37 male, 39 female): five FGs of first year of Compulsory Secondary Education, five FGs of fourth year of Compulsory Secondary Education and four FGs of first year university students.

#### 3.1.1 Procedure

The focus groups were designed with semi-structured prepared questions and topics and were conducted and audio-recorded in the settings with the consent of the parents or guardians of participants under 18 and of the students themselves. The open-ended script is based on the prior literature review (Buckingham, 2008; Ferrés and Piscitelli, 2012; boyd, 2014; Gill, 2017; Aran-Ramspott et al., 2018, 2022; Scolari, 2018), the method (Barbour, 2007) and the objectives of the study. The script, which was intended to be flexible as this was an exploratory analysis, initially canvassed general categories related to the participants’ views of social media, their preferences and motives for consuming social media; types of functions and uses made; characteristics of preferred YouTubers and Instagrammers; and specifically, toward the participants’ identification and perception of (trans)media competencies in Ideology and Ethics [Aran-Ramspott et al., 2024; based mainly on Ferrés and Piscitelli (2012) and Scolari (2018)]. These competencies can be summarized as the ability to detect and critically analyze representations of stereotypes related to gender, or sexual and gender orientation, among other things, and the ethical and social implications related to processes of emotional identification, manipulation or invisibility of certain groups, including women. Gill (2017) particularly allows us to review notions seen in contemporary culture as “postfeminism” which currently operates as a kind of gender neoliberalism (“cultivation of the “right” dispositions to survive in neoliberal society: confidence, resilience and self-confidence”).

The open-ended script design facilitated the coding of categories; new categories emerged from participants’ comments. First, the literal comments (in sentences or phrases) related to the priority dimensions of the script were independently analyzed by each researcher. Second, they were cross-checked within the team to refine and validate the coding in successive sessions (Bryman, 2012), taking into account their nature and importance, and in light of their frequency, occurrence and repetition. In order to gather data on participants’ gender perspectives, the relevant interventions were taken to be those in which an informant reflected their personal gender perspective in the discourse in relation to social media. We analyzed co-occurrence with the type of social media, content, reasons for use, references to influencers, (trans)media competence and characteristics of the remarks by age and gender (in function of how each participant introduced themselves). The location variable proved not to be significant in the focus groups. Coding shows the FG number _age_gender (F: Female/M: Male, the numbers show order of intervention where there is more than one participant of a given gender).

The research was approved by the Ethics Committee of University Ramón LLull University, Barcelona, which confirmed that participation was voluntary and anonymous, the confidentiality of the participants’ data, the collection of permissions and informed consents. This research was overseen and monitored by the public body that financed the research project.

## 4 Results

**Figure 1:**
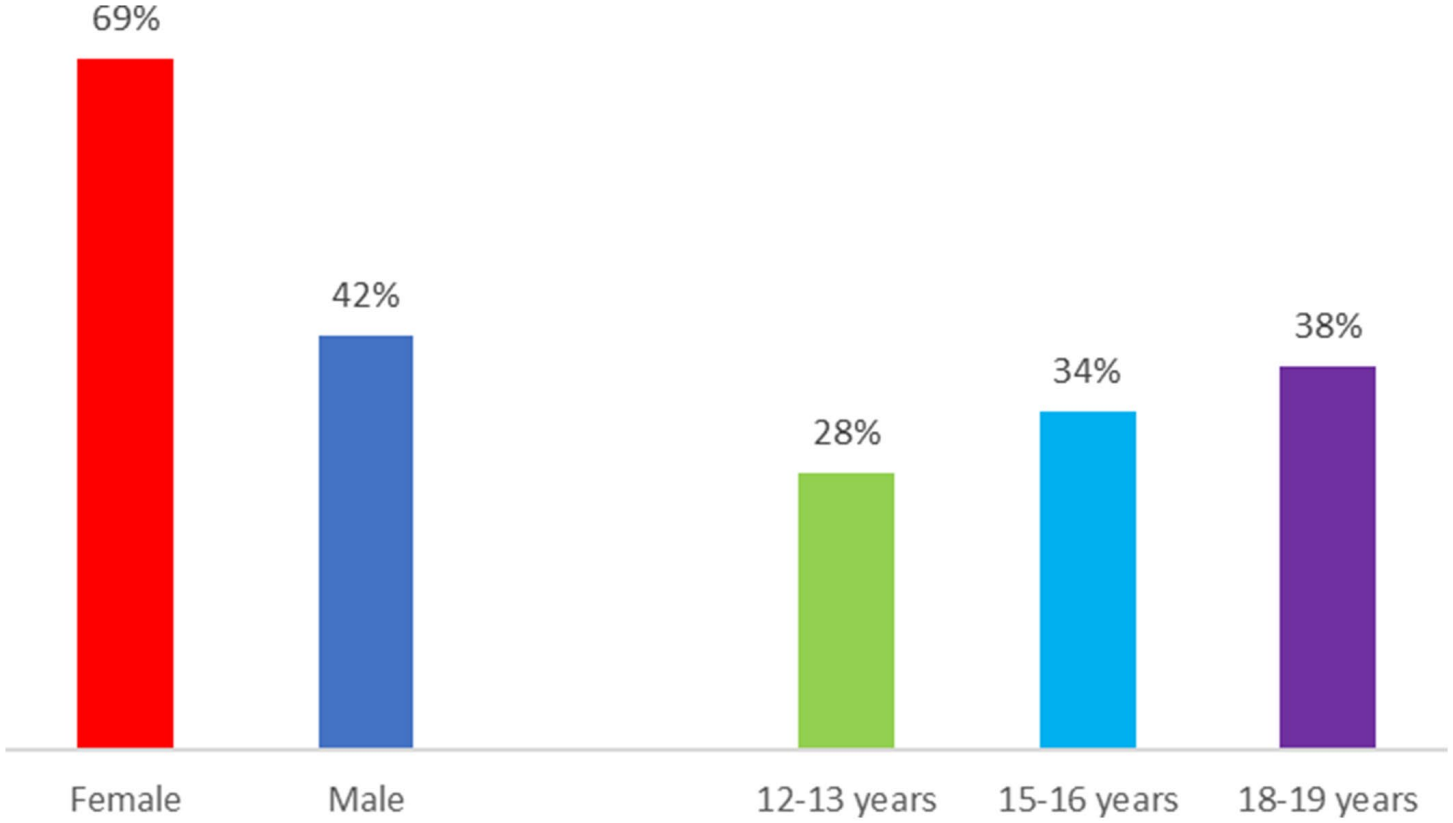
Distribution by gender and age of comments concerning gender perspective.

**Figure 2:**
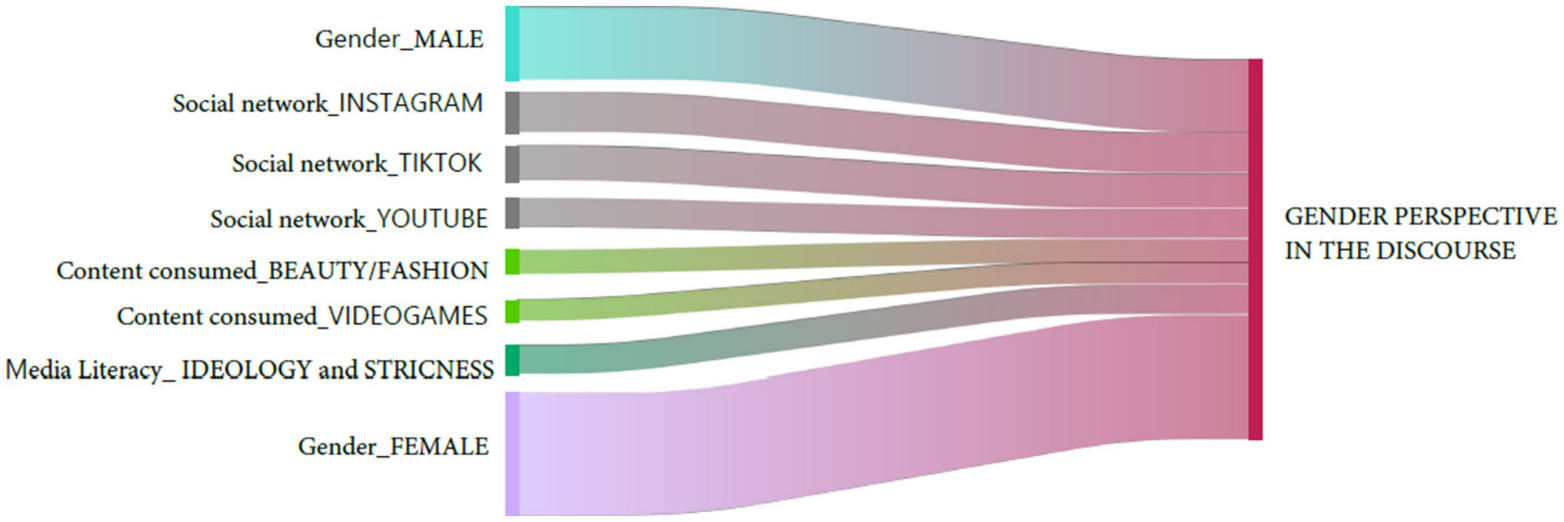
Principal co-occurrences of the gender perspective by dimension (Sankey Diagram).

The most relevant results directly related to the research questions for the study are presented below. First, we show distribution by gender and age of the total number of comments by participants in the FGs in relation to gender perspective in their discourse in relation to social media (Figure 1), so as to show the origin of each remark. Figure 2 shows principal co-occurrences of the gender perspective in the discourse in relation to the dimensions set out in the materials and method section.

Explicit mentions of gender perspective have been broken down by gender of the participants. As can be seen in Figure 1, female participants made almost 70% of the total number of comments. By age, the gender perspective is mentioned progressively more at older ages: 27.7% among 12–13 year-old participants (1st year of Compulsory Secondary Education), 33.8% in 15–16 year-olds (4th year of Compulsory Secondary Education) and 38% among 18–19 year-olds (first-year university students).

Figure 2 shows not only how female participants comment materially more on the gender perspective, but also how the information from the focus groups identifies the dimensions that students consider more important. Thus, dimensions related to the type of social media, the type of content, the definition of influencer, (trans)media skills and age are more salient in participants’ discourse. Further detail is provided in the following paragraphs.

### 4.1 Participants’ perception of gender perspective by type of social media

The analysis of the perceptions of adolescents and young people of gender on social media shows that most comments concern Instagram (23%), TikTok (20%), and YouTube (17%). TikTok was spontaneously mentioned by participants. In terms of gender, females make more mentions than males, and refer to Instagram, TikTok and YouTube in descending order.

But what is the discourse of young people as revealed by these comments about social media? In relation to Instagram, TikTok and YouTube, the significance attributed to personal appearance is what is most commented on. The obsession with physical appearance is expressed in terms of self-representation, whereby appearance contributes to identity in terms of approximation to esthetic ideals and beauty standards:

“Lots of girls who go on TikTok may be affected by seeing influencers who look perfect, at least at first, and then they may look at other bodies and that may lower their self-esteem” (G9_12–13_F).

The same is true of the sexualization of physical appearance, which participants refer to, directly or indirectly, in relation to the bodies of girls and women. Instagram is perceived to be somewhere where females can more readily take a leading role.

“Boys are more popular on YouTube and on Instagram it’s women, perhaps girls’ content seems more attractive, not only because it is more or less sexualized, which depends, but because on Instagram you have to be really careful, you have to know what photo to take, how, what lighting, see how you tell the story, whether you post it this way or that way, and girls take greater care over those details, and so they are more popular” (G14_15–16_F).

Females make almost twice as many comments about the importance of body image as males (21 and 13 quotes, respectively):

“I think as girls we expose ourselves more” (G4_18–19_F).

In turn, they are more affected by beauty standards, including the risk of developing an eating disorder.

“[Referring to the influencer Marina Yers]… but to say that after eating, being sick is good for you… That can lead to illness in 12-year-old girls” (G2_15–16_F).

On the other hand, there are female participants who demand parity with males in relation to the display of the body on the social media, with comments that identify contradictions inherent to post-feminist discourses (Gill, 2017), by presenting as a personal choice what others might see as the internalization of objectification or sexual neoliberalism (de Miguel, 2015).

“TikTok also does a lot of harm, for example if any of the three of us posts a video of us wearing a top or bikini, TikTok unpublishes it, it does not let us post it, but the guys can post videos in their underwear or shirtless, and TikTok’s algorithm does not say anything to them. We are super censored (G10_15–16_F).

-And on Instagram even more so (G10_15–16_M).
- No (G10_15–16_F2).
- More on Instagram than TikTok (G10_15–16_M).
- I posted a video in trackies and T-shirt and they censored it” (G10_15–16_ F3).

YouTube is seen as a social media platform on which people can find less invasive spaces and activities supportive of the manifest need to improve self-esteem and build a more empowered attitude. On two separate occasions, 12-year-old girls explicitly mention an empowering movement, “Love in positive” and, from a feminist perspective, “Me too.”

“I have watched a YouTuber who I think is a good influence, there are a lot of people now who say they feel insecure because of their body, but this influencer was about “Love in positive,” love yourself as you are, and I think that a very good thing about social media is people like that who are trying to boost your self-esteem, make you feel confident” (G11_12–13_F).

Self-confidence, related to satisfaction with a person’s own body, is shown on social media through the control of image, posture, and esthetics. Young people subject themselves to those mechanisms depending on what is trending. No comments were collected, however, in which male participants acknowledge the influence of social media on their image. In contrast to females, their attitude appears distanced or rebellious.

“I like to be happy, and fashion is the last thing that interests me, for example, if everyone is wearing Nike, I’ll still wear Adidas (…) I’m fine with my 80s hairstyle” (G10_15–16_M).

### 4.2 Participants’ perception of gender perspective by type of content consumed

Generally speaking, the discourse of both male and female participants shows that the content consumed is highly gender specific.

“Do boys and girls follow the same things at this age? (Moderator 1, G1_12–13).

- No (G1_12–13_F&M).
- Some things Yes and some things No (G1_12–13_F).
- Sometimes maybe, what we have most in common is series, films…(G1_12–13_F2).
- Him and me do not share any content, he’s more into sports and I’m more into languages, travel…” (G1_12–13_F).

Comments about entertainment in terms of watching series and films come equally from male and female participants. There are differences in which types of content are most frequently mentioned: Beauty/fashion and Videogames. Dance is only commented on by male participants (“usually the dances on TikTok are by girls”), in contrast to current affairs. This confirms earlier results on gender specific preferences for content types (Fernández-de-Arroyabe-Olaortua et al., 2018; García-Jiménez et al., 2021), and about motivation, where boys and young men express a preference for entertainment (Lozano-Blasco et al., 2023). In this study, entertainment and strengthening friendships are positively rated by both genders. For example:

“(On Youtube), but I think my friends are more on Instagram, they make stories and I see what happened to them during the day, we talk and you can also go to the entertainment area…” (G11_12–13_M).

### 4.3 Participants’ perception of gender perspective by influencer

Basque streamer Ibai Llanos is most recognized for his positivity – acknowledging his personal and financial interests – and for his friendly personality. Consequently, he is the influencer who has the most followers, both male and female.

“And we are talking about YouTube, Instagram and TikTok, which are, like, social media, at the end of the day kids are searching for tutorials on how to play Minecraft, they are searching Ibai, because a lot of boys, and some girls of course, are obsessed with Ibai. I see Ibai as everything, super-responsible and very incisive. There are many others who aren’t, but Ibai just is. At the end of the day, if you upload content, you are going to influence those children through that content” (G6_18–19_M).

The participants associate the term influencer more with women who are successful on social media (Dulceida and Paula Gonu, two Catalan bloggers seen as celebrities, clearly figure in that role in the comments). The association between girl or woman and influencer is much more apparent as perceived by the participants, and is attributed to fame (celebrities, It Girls) and to so-called posing, which does not seem to apply to boys or young men.

“There are also influencers, boys, (…) but I have not seen a boy say he is an influencer, or that he uploads that type of content” (G6_18–19_F).

Some girls, especially older girls, explicitly express pleasure in displaying themselves, in line with the style of some influencers. For instance:

“I really like influencers, seeing how they dress, to keep up to date with fashion and I also really like to display my life, especially for the people around me to see what I do, I like them knowing about it” (G4_18–19_F).

At the opposite end of the spectrum to popular influencers, there is universal criticism of Naim Darrechi, a TikToker from Mallorca, known for his controversial sexist remarks, which have even led him to be accused of seeking to justify rape and sexual abuse.

“He is a sexist and has done a lot of bad shit” (G7_15–16_ M).

“There are people who follow him and the worst thing is that he’s proud of what he says and even has a lot of followers” (G2_15–16_F).

### 4.4 Participants’ perception of gender perspective by transmedia competence

The vast majority of comments linking the gender perspective to transmedia competencies (Scolari, 2018) refer to the dimension of Ideology and Rigorousness, which by definition includes stereotyping, emotional identification mechanisms and recognition of manipulation (e.g., fakes). Comments, especially from female participants, reflect participants’ awareness of the need to (self-)regulate their media diet and of the risk of becoming addicted, even though a number of participants, particularly younger participants, explicitly state that they have been given cybersafety training at school.

“Right, I think it’s the typical thing that you say to everyone “Instagram is bad, do not be on it so much”, but I know that there are things that you cannot watch and I do not watch them, but my parents aren’t on my case the whole time” (G1_12–13_F).

The rest of the (trans)media competencies identifiable in the discourse of the young participants from a gender perspective were only commented on by the female participants. In descending order, comments fall under Language and Esthetics, Technology, and Production and Dissemination, where female participants comment on aspects such as the algorithm used.

“It’s that TikTok gets you addicted, you say you are going to watch it for 5 min and then a whole day goes by (…) with Instagram it’s harder to get addicted, because on TikTok you scroll down and more and more videos come up, and on Instagram there’s a moment when there’s nothing else to see” (G1_12–13_F2).

### 4.5 Participants’ perception of gender perspective by age

In the results, the gender perspective also appears to be related to age, expressed as a concern for children.

“There are a lot of 11 and 12-year-old girls (…) most of them are girls (…) who join a fan club, whatever. And they do not stop to think what nonsense the people are talking. And I think so much freedom has been given to those content creators that they think they have the right to publish whatever they want, without thinking that their fame is due to people who aren’t mature enough to think and weigh opinions, and to see what’s right (…) your followers are at an age when if you tell them something, they’ll believe you, it’s like you are indoctrinating them, they are so young that they’ll believe anything you say” (G10_15–16_M).

## 5 Discussion and conclusions

The presence and use of social media by young people requires a debate in wider society and the academic community around the opportunities and risks that social media present in the construction of young people’s identities. Of particular importance is the analysis of the gender perspective that emerges in the context of digital empowerment, since it reflects the expression by young people of differing levels of awareness of their responsibility for progress toward a just society, free (among other things) of gender-based prejudice and inequality.

In relation to the first research question, whether young people of both sexes consider gender in their assessments of social media – such as YouTube and Instagram -, the results show that it is principally females who refer to gender in their comments. They associate gender with the importance of image and appearances on social media, principally on Instagram and TikTok. Our results are partially consistent with a focus group study with 15 to 19-year-old teenagers in Finland (*N* = 35) and provided understanding of the important role of commercial social media in young people’s consumption styles. While in the Finnish study boys appeared more materialistic and interested in luxury and sustainable consumption seemed to be a more “girly” thing (Wilska et al., 2023), in our research boys do not acknowledge the influence of social media on their image. Female participants perceive that they are more exposed and under greater pressure than boys and young men, a perception also reflected in the comments of male participants. They also associate that pressure mostly with fashion and, to a lesser extent, with trends in consumer or capitalist society. Among male participants, there are more expressions of self-assertion and self-judgment. Both genders express concern about the effects of this body-image pressure on young girls. There is no mention of other possible gender identities beyond male and female (non-binary, gender-fluid…).

The obsession with image as a way of making visible the self is part of a generational culture of appearance and public approval (Guardiola, 2018), which is acknowledged with critical reflection by both male and female participants. Sexualization is associated with certain influencers.

In relation to the second research question, whether the perception of male and female social media users can be seen as empowering for them, most of the positive comments from both genders in discourse concerning YouTube and Instagram refer to entertainment and the strengthening of friendships and the sense of belonging to a peer group. This feeling of belonging, which is reinforced by influencers, can be recognized in a small-scale qualitative research work in Germany: adolescents are often particularly attracted to influencers they believe themselves to have things in common with, such as around gender/sex, hobbies, and geographical location [Bamberger et al., 2022, cited in Pérez-Torres et al. (2018), Diepeveen (2024)].

The results allow us to identify comments that include some sensitizing concepts (Gill, 2017) articulated around the construction of gender on social media, such as comments on the “Me too” movement. However, as noted, the importance of image is mentioned to a greater extent by female participants and in relation to material on social media directed at girls and women, while male participants particularly mention having fun and success or social status. Explicit references to self-esteem are also more common among female participants, especially among young girls, in the form of concern about low self-esteem due to the influence of ideal bodies, as often displayed by influencers. In fact, there are no comments from male participants that acknowledge the influence of social media on their self-image, rather the reverse. This may suggest less external oversight and greater self-confidence than among female participants. The spontaneous expressions of such views may reflect the internalization of personal empowerment among male participants.

On the other hand, some of the youngest girls (aged 13–14) demand fairer treatment on social media and that demand is based on the rejection of censorship of their bodies by social media platforms. As Caballero-Gálvez et al. (2017) point out, we can reconcile the apparent paradox of freedom of choice and the forcefully expressed demands of young girls for the recognition of an (idealized) image rather than gender equality. In relation to the characteristics of postfeminist discourse (Gill, 2017), we saw this emphasis on bodily self-monitoring, especially among female participants, related to the management of the individual’s own image and perceived sexualization in some representations.

The contribution of this study is to attend to the voices of adolescents and young people, providing them with a platform to express their views freely in focus groups. This approach offers valuable insights into their perceptions and experiences with social media and gender. Focusing on young people’s perception of gender issues on social media is a critical additional layer in the discussion around the potential consequences for young adults and adolescents of content, use and engagement in social media. For adolescents, social media constitute a new arena in which they can express themselves and explore, but they are also to a significant forum for the dissemination of certain beauty standards that may represent a risk to adolescents in relation to their body image (Arab and Díaz, 2015; Segovia Aguilar et al., 2016; Malo-Cerrato et al., 2018; Shah et al., 2019). One can find on social media content related to the human body that may influence adolescents and cause them to obsess about their appearance and the photos that they post (Goodyear, 2020). In that sense, the risk resides in interiorization of such messages, especially by girls, which may have significant repercussions on their body image and mental health (Fredrickson and Roberts, 1997; Karsay et al., 2018). Given differences in gender roles and societal pressure, there is a need to display an image that society considers acceptable. In that sense, social media and mass communication media play a fundamental role in objectification, and may have a significant impact on self-esteem, self-image, body positivity and psychological wellbeing (Aydm and San, 2011; Shah et al., 2019). In short, this research shows how the digital empowerment of young people is constructed through the dominant paradigm of image in its presence and representation on social media.

However, from a gender perspective, female participants perceive the negative dimension of this (false) empowerment more than males. Across social media platforms, as also noted by Scharrer et al. (2023), our results show that YouTube is perceived to give more equal prominence to the genders and to have more balanced activities and content suitable for each gender relative to Instagram and Tiktok. In our results, the impression predominates that YouTube is where males contribute more content. On Instagram, on the other hand, the importance of body image is clearly recognized by young people, especially females, in consequence of the centrality of (self-)image on Instagram, which in turn reflects the logic of today’s society of (hyper) visibility (Imbert, 2004).

Finally, we have identified tensions that could even be seen as contradictions in the perceptions of young users of social media, in relation to life offline. The blurring of the boundaries between intimacy and extimacy may be a cause for more hope than might appear: Sabich and Steinberg (2017) believe that, although the discourse of YouTubers is trapped in a consumer culture, those online spaces for interaction allow the construction of symbolic bonds of belonging, that are particularly propitious for young people.

“The evidence indicates there is not a simple cause and effect relationship between social media use and (harmful) gender attitudes” (Diepeveen, 2024, p. 9). Part of the context for our research is the fact that the relationship between use of social media and gender equality is not simple and involves at least three different elements: personal experience and social context, i.e., individual perceptions; platform design, and online experience, especially the types of relationship built with social media (Diepeveen, 2024). Our study highlights different aspects in relation to those different elements, based on the perceptions of our young participants. The limitations of our study include that the sample was drawn from a specific geographic area and culture in Spain. Some prior research in Spain (e.g., García-Jiménez et al., 2021; Herrero-Curiel and La-Rosa, 2022; Serrate-González et al., 2023) studied the behavior of adolescents on social media in general, “but so far as we have been able to confirm there has been no research looking at their preferences in relation to the content generated specifically by their favorite influencers” (Martín-Cárdaba et al., 2024, p. 83). Another limitation is that the study coincided with the Covid-19 pandemic. This exceptionality meant that the importance of social media was increasing (Wilska et al., 2023).

The results of our research are consistent with other works in expressing concern about gender differences that go beyond differences in degree of participation and topics engaged with on social media. Our data align with findings in Europe [EU Kids online 2020 in Smahel et al. (2020)] that show that the digital gender divide does not reflect a significant difference among European youth in terms of access. It rather betokens prevalent differences in the modalities of use and consequently in the skills deployed (Masanet et al., 2021). Processes of socialization reflect the structural inequality fostered by, among other agents, the media system itself. That seems to be a persisting historical issue rather than a contemporary anomaly and entails a so-called cognitive cost for the most disadvantaged, including women (Benesch, 2012).

From a developmental perspective, here we take up Kabeer's (1999) idea of trajectory, describing a notion of transmedia education that engages with new technologies and social realities, with empowerment as an individual process leading to greater autonomy and capacity to make life choices; and as a collective process of the development of a group’s capacity to drive social change so as to create a just, fair society, particularly in terms of relationships between men and women (Charlier and Caubergs, 2007, p. 6). In other words, empowerment here means achieving not power over but power to, power with and power within, as described by those authors (Charlier and Caubergs, 2007, p. 10).

The fact that the study’s sample was drawn from three Autonomous Communities increases the diversity of perspectives and enriches the data collected. While it limits the extent to which the findings can be generalized to other cultural or geographical contexts, it opens avenues for future research.

Despite the limitations of extrapolating qualitative data to adolescents and young people in Spain more widely, this study allows us to hear the participants and so develop greater understanding of digital empowerment from an edu-communication and gender perspective. The interpretation of the results points to the need for transmedia education that promotes reflection and critical production among adolescents and young people, specifically from the perspective of their as-yet only partially constructed identities that pays greater heed to the importance of aspects of their lives beyond conventional ideas of physical beauty. In that area, users need to become more aware of the influence of social media in the construction of notions of gender and to develop their capacity to engage critically with such notions in both formal and informal settings. That will enable them to engage with postfeminist perspectives in the context of the dominant values of today’s neoliberal society (Medina, 2021), particularly as concerns the internalization of rigid beauty standards in relation to the bodies of women and girls especially, through (for example) programs to make people more aware of their own stereotyped beliefs around gender (Panerati et al., 2023). The journey is for both boys and girls, men and women, toward greater empowerment of women and girls and a respectful, fair system that embraces diversity.

Although this study acknowledges gender disparities in social media, to delve deeply into the potential causes or solutions would strengthen the impact of future works. The evolving nature of social media platforms and research with “big data” in the field of perceptions and sexist content is still limited, not only because of the role of algorithms, but also because of aspects such as pseudonymization (Lozano-Blasco et al., 2023) or because of adolescents’ own search for an ideal representation of themselves (Stockdale and Coyne, 2020). Moreover, adding a comparative analysis with older age groups or with data from other countries could offer a broader perspective on how these perceptions might vary across different demographics or cultural contexts.

Based on our findings, we provide specific considerations for educators, policymakers, and social media platforms on how to address the identified issues. Furthermore, future works should develop, implement and evaluate edu-communicative and media education initiatives to develop critical reflection on the influence of social media, given that the results of this study underline the importance of a respectful and equitable environment in relation to gender equality in digital spaces, which helps to implement educational measures for working with esthetic and erotic body image (Pires et al., 2021), to develop self-awareness and self-regulation (Mirgos et al., 2023) and to deepen protective factors against the risks online (Ramos-Soler et al., 2018). Along those lines, the sessions focused on the responsible critical use of the Internet and digital devices for adolescents aged 13–15 developed by Cuervo et al. (2022a), and aged 12–17 by Medrano et al. (2019) and Cuervo et al. (2022b); the classes put forward by Mirgos et al. (2023) around hate speech, privacy, digital intoxication, and perceived values on social media; the digital interactive tool to develop and assess the media competence of European students aged 14–18 (Ferrés et al., 2022); the worksheets in Scolari (2018); and the use of Service Learning proposed by Villacampa et al. (2020) for collaboration to eradicate gender violence through online behaviors deserve special attention. Moreover, we consider that in this process both teachers and families should be involved together with students, to educate in the ethical and responsible use of social networks, for example using the guides developed by Martínez Ten (2021).

As discussed above, future works should address the role of digital platforms in highlighting stereotypical representations of men and women (Bernárdez, 2015) could lead people to internalize inequality, due that evolving nature of social media platforms and research with “big data” in the field of perceptions and sexist content is still limited.

Policy makers should consider the importance of promoting a respectful and equitable environment in relation to gender equality within digital spaces, in order to provide support for the need to develop and implement edu-communicative initiatives to foster critical thinking around the influence of social media in this context and the evaluation of the impact of such initiatives in future research.

## Data availability statement

The anonymised raw data supporting the conclusions of this article will be made available by the authors, without undue reservation.

## Ethics statement

The studies involving humans were approved by the Research Ethics Committee of the project’s coordinating University (Blanquerna School of Communication and International Relations, Ramon Llull University, Barcelona, Spain). The studies were conducted in accordance with laws applying throughout Spain and locally to the research institutions and the requirements of those institutions. Written informed consent for participation in this study was provided by the participants and/or their legal guardians/next of kin. Online written and paper written informed consent was obtained from the individual(s) and/or participants’ legal guardians/next of kin for the publication of any potentially identifiable data included in this article.

## Author contributions

SA-R: Writing – review & editing, Writing – original draft. OK-A: Writing – review & editing, Writing – original draft. IE-A: Writing – review & editing, Writing – original draft. ÁM-I: Writing – review & editing, Writing – original draft. IB-G: Writing – review & editing, Writing – original draft.
